# A manual method to obtain platelet rich plasma

**DOI:** 10.1590/1413-78522014220200798

**Published:** 2014

**Authors:** Fabiana Paulino Marques, Sheila Jean McNeill Ingham, Andrea Forgas, Carlos Eduardo da Silveira Franciozi, Pedro Henrique Sasaki, Rene Jorge Abdalla

**Affiliations:** 1 Hospital do Coração , Knee Institute, São Paulo, SP, Brazil, Knee Institute, Hospital do Coração (HCor), São Paulo, SP, Brazil; 2 Universidade Federal de São Paulo, Escola Paulista de Medicina, Department of Orthopedics and Traumatology, São Paulo, SP, Brazil, Department of Orthopedics and Traumatology, Escola Paulista de Medicina, Universidade Federal de São Paulo, São Paulo, SP, Brazil

**Keywords:** Platelet-rich plasma, Blood platelets, Methods.

## Abstract

**OBJECTIVE:**

This study is to report a manual method to obtain platelet rich plasma (PRP).

**METHODS:**

For this study 61 ml of peripheral blood was obtained and submitted to centrifugation at 541g for 5 min. The centrifugation separates the blood into three components: red blood cells, buffy coat and platelet rich plasma. Blood and platelet rich plasma samples were sent to the Hospital's Laboratory and platelets and leukocytes were measured.

**RESULTS:**

A sample of 637 blood donors was evaluated. The platelet yield efficiency was 86.77% and the increase in platelet concentration factor was 2.89 times. The increase in leukocyte concentration factor was 1.97 times.

**CONCLUSION:**

The method described here produces leukocyte-rich and platelet-rich plasma with a high platelet and leukocyte increased factor. ***Level of Evidence IV, Controlled Laboratory Study.***

## INTRODUCTION

Platelet-rich plasma (PRP) was first described by Whitman *et al*.v hrhh^1^ in 1997 as a derivative of fibrin glue made ​​by Matras,^2^ and today its use has been widely documented in the medical and dentistry literature.^3^ ^-^ ^5^ Dental surgeries, plastic surgeries, as well as orthopedics, have shown good results with the use of PRP to obtain better healing.^3^ ^-^ ^12^ Although most reported positive results, there is no conclusive evidence of the effect of PRP on tissue healing^13^ and one of the reasons could be the lack of knowledge of the basic characteristics of PRP, as the number of platelets required, and the need for activation of these platelets.^3^

PRP can be defined as a fraction of a volume of plasma that has a higher concentration of platelets than in peripheral blood.^14^ ^,^ ^15^ Platelet concentration and amount of growth factors in the PRP depend on the technique used,^16^ but on average, PRP has 3-5 times more growth factors than peripheral blood.^17^ Today there are several techniques to obtain PRP and this has led to confusion regarding the classification,^17^ ^-^ ^19^ the time and the centrifugation speed, which are extremely variable.^18^

The use of PRP for tissue regeneration has grown, but it still needs further research and clarification regarding methods of its obtention^20^. The aim of this study is to demonstrate a new manual method of obtaining PRP.

## MATERIALS AND METHODS

This study was approved by the Ethics Committee and was conducted in accordance with the ethical standards established by the Helsinki Declaration of 1964. All subjects who underwent knee surgery at our institution who received PRP were included in this study. All subjects gave informed consent before inclusion in the study. Data were collected from 2008 to 2010.

**Figure 1:**
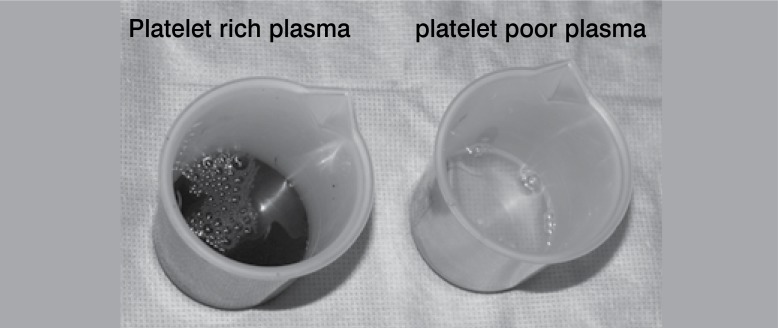
Platelet poor plasma (right) and platelet rich plasma (left).

**Figure 2:**
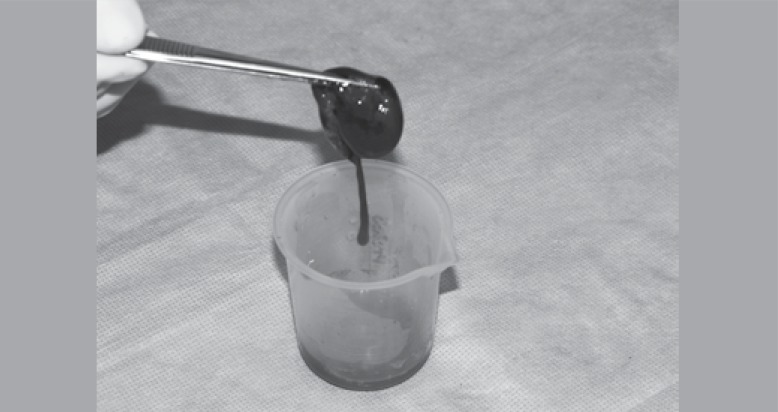
Platelet rich plasma clot activated with autologous thrombin and 10% calcium gluconate.

For this study 61 ml of peripheral blood was collected from each patient. One milliliter was used to count the number of platelets and leukocytes in the peripheral blood and 60 ml were used to obtain PRP. Five milliliters of the anticoagulant sodium citrate were used and centrifugation at 541g for 5 minutes (Centribio 80-2B centrifuge Centribio, São Paulo, SP, Brazil) to obtain 18ml of PRP and 14ml of poor platelet plasma. (Figures 1 and 2) Centrifugation separates blood into three components: red blood cells, buffy coat and PRP. PRP and buffy coat are carefully collected to prevent any contamination with red blood cells.

Samples of whole blood and PRP were sent to the hospital laboratory and platelets and leukocytes were quantified with a Sysmex - XT1800i hematology analyzer (Sysmex America, Inc., Mundelein, Illinois).

## STATISTICAL ANALYSIS

Statistics data was calculated using a statistical software SPSS version 17.0 (SPSS Inc., Chicago, IL, USA). The Wilcoxon test was used to compare values pre-and post-centrifugation. The Mann-Whitney and Kruskal-Wallis tests were used to compare the difference between groups. Dunn's multiple comparison was used as *post hoc* test. Data are presented as mean ± standard deviation. Statistical significance was set at 0.05.

## RESULTS

A sample of 637 subjects was evaluated. Of these, 637 had the number of platelets in peripheral blood quantified, and 445 had the number of leukocytes quantified in peripheral blood and in PRP. The mean age was 45.78 ± 15.11 years old and 75% were male.

The mean platelet count and leukocytes in peripheral blood was 220.377 ± 51.484/mm^3^ e 7.149 ± 4.777/mm^3^, respectively, while the number of platelets and leukocytes in PRP was 637.388 ± 189.962/mm^3^ and 14.056 ± 11.820/mm^3^ (p < .001 for both).

The efficiency of platelet capture^21^ (PRP volume x [platelets PRP] / (blood volume x [blood platelets]) was 86.77% and the increase in the concentration of platelet factor was 2.89 times. The increase in the leukocyte concentration factor was 1.97 times.

In males, the mean platelet count in whole blood was 214,184 ± 49.732/mm^3^ and in PRP it was 626,718 ± 191.917/mm (p < 0.001) whereas in females these values were, respectively, 238.994 ± 52.327/mm^3^ and 669.465 ± 180.778/mm^3^ (p < 0,001). The difference between genders was statistically significant for counts in whole blood (p < 0.001) and for PRP count (p=0.005).

Table 1 shows the distribution of platelets and leukocytes, divided by different age groups. *Post- hoc* analysis using Dunn's multiple comparison showed differences in the following groups: 

**Table 1:** Distribution of platelets and leukocytes in whole blood and in PRP according to donor's age.

Variable	Age (years old)	Median	Minimum	Maximum	N	p
Platelets in peripheral blood (platelets/mm3)	< 20	224.000	129.000	283.000	20	0,032
	20 - 29	226.000	125.000	363.000	83	
	30 - 39	217.500	139.000	335.000	122	
	40 - 49	226.500	112.000	412.000	148	
	50 - 59	210.500	107.000	364.000	136	
	60 - 69	208.500	127.000	393.000	84	
	> = 70	202.000	101.000	346.000	39	
Platelets in PRP (platelets/mm3)	< 20	597.500	429.000	835.000	20	0,199
	20 - 29	627.000	216.000	1.178.000	83	
	30 - 39	604.500	248.000	1.156.000	122	
	40 - 49	636.000	304.000	1.615.000	148	
	50 - 59	621.500	203.000	1.229.000	136	
	60 - 69	580.500	301.000	1.686.000	84	
	>= 70	644.000	273.000	1.090.000	39	
Leukocytes in peripheral blood (leukocytes/m3)	< 20	6.555	4.610	9.000	12	0,001
	20 - 29	6.810	4.090	15.280	57	
	30 - 39	7.100	4.290	15.960	87	
	40 - 49	6.880	3.700	79.990	114	
	50 - 59	6.230	2.660	9.360	95	
	60 - 69	6.500	4.200	14.860	57	
	> = 70	6.150	3.700	66.600	28	
Leukocytes in PRP (leukocytes/m3)	< 20	12.010	9.800	17.100	12	0,002
	20 - 29	14.100	3.960	36.040	63	
	30 - 39	13.010	3.250	39.860	95	
	40 - 49	13.700	3.830	209.020	118	
	50 - 59	10.830	4.110	28.500	102	
	60 - 69	12.550	4.440	32.200	66	
	> = 70	11.200	5.500	140.700	28	

a) Platelets in whole blood: 20-29 *vs.* 50-59 (p = 0,022), 20-29 *vs.* > = 70 (p = 0,026), 40-49 *vs.* 50-59 (p = 0,007), 40-49 *vs.* 60-69 (p = 0,027) e 40-49 *vs.* > = 70 (p = 0,017); 

b) Leucocytes in whole blood: 20-29 *vs.* 50-59 (p = 0,03), 30-39 *vs.* 50-59 (p < 0,001), 30-39 *vs.* 60-69 (p=0,043), 30-39 *vs.* > = 70 (p = 0,017) e 40-49 *vs.* 50-59 (p = 0,003); 

c) Leucocytes in PRP: 20-29 *vs.* 50-59 (p < 0,001), 30-39 *vs.* 50-59 (p = 0,015) e 40-49 *vs.* 50-59 (p < 0,001).

## DISCUSSION

This study demonstrates the variability in the number of platelets and leukocytes in peripheral blood and PRP in a large population. We also show that the method described herein shows an increased concentration factor of platelets and leukocytes, and that there is high platelet collection efficiency. We also demonstrated significant difference in platelet count when comparing different ages and gender, since females showed a significantly higher amount than men, and younger people showed higher platelets and leukocytes count.

**Figure 3:**
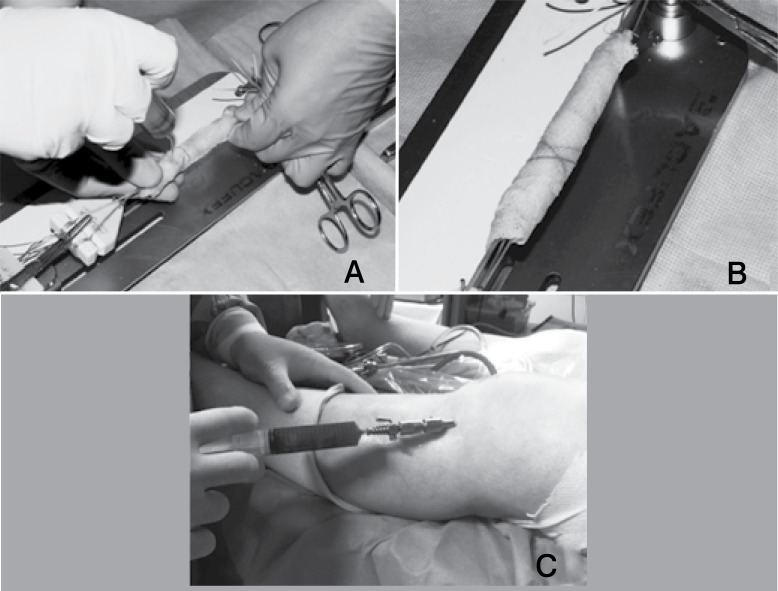
Examples of clinical application of PRP: A) Placement of PRP in autograft to be used for reconstruction of the anterior cruciate ligament; B) Soaked autograft wrapped in gauze soaked with PRP. C) Application of PRP after knee arthroscopy.

The use of growth factors as a stimulus for tissue healing has been studied in several areas of orthopedic and dentistry surgery.^3^ ^-^ ^5^ ^,^ ^22^ In Orthopedics, it has been successfully used in bone soft tissue healing procedures, in the reconstruction of the anterior cruciate ligament rupture, in Achilles tendon rupture in athletes, and in surgical scar after total knee arthroplasty.^3^ (Figure 3) Nevertheless, we have no conclusive evidence on the effect of PRP on the results obtained,^3^ and we do not know yet the best method for PRP application.^6^

A previous study from Castillo *et al*.^21^compared three automated methods to obtain PRP. The highest platelet capture efficiency was obtained with Cascade MTF, and it was of 67.6%, a value lower than our study's (86.77%). This may be due to the large volume of the obtained PRP (18 mL). Moreover, we had a high incremental factor, showing that this manual method may be used to obtain PRP. Obtaining PRP by automated methods is expensive,^23^ and can be prohibitive in developing countries, like ours. The use of a manual centrifuge, available in most hospitals and surgical centers, can make this method more available and ready to use. Thus, PRP can become a cheaper source of growth factors (PDGF, TGF-β, VEGF, IGF-1, etc.) and can stimulate the tissue healing.^6^ ^,^ ^7^ Castillo *et al*.^21^ also measured the amount of white blood cells and found no difference between whole blood and PRP. This result is different from ours, since we found significant differences (p < 0.001) for leucocytes. This may be a result of the manual method used in this study, where the buffy coat was intentionally included in the preparation of the PRP. A highest concentration of leukocytes can lead to a higher concentration of PDGF (platelet growth factor)^11^and this is an important growth factor for tissue regeneration, since it is a potent stimulator of mesenchymal cells (fibroblasts, smooth muscle cells) mitogenesis^24^ in addition to stimulating angiogenesis and macrophage activation.^15^ The presence of leukocytes may increase the anti-microbial activity of PRP as well as analgesia.^19^

There is still much confusion regarding the classification of PRP. Dohan Ehrenfest *et al.* ^18^ suggested the following classification: leukocyte poor PRP; platelet and leukocyte rich plasma; platelet rich and leukocyte poor fibrin; and platelet and leukocyte rich fibrin. We believe that PRP obtained by the method described in this study is the platelet and leukocyte rich plasma, as there was a large incremental factor for both, platelets and leukocytes.

## CONCLUSION

The method described herein produces platelet and leukocyte rich plasma with high leukocyte and platelet incremental factor.
